# Supplementary material for: Reducing methylation of histone 3.3 lysine 4 in the medial ganglionic eminence and hypothalamus recapitulates neurodevelopmental disorder phenotypes
Source: Nat Commun. 2026 Feb 20;17:2984. doi: 10.1038/s41467-026-69248-9 (PMC13035845; doi:10.1038/s41467-026-69248-9)
Supplement: Supplementary file 1 — Supplementary Information [file 41467_2026_69248_MOESM1_ESM.pdf]

Supplementary Figure 1

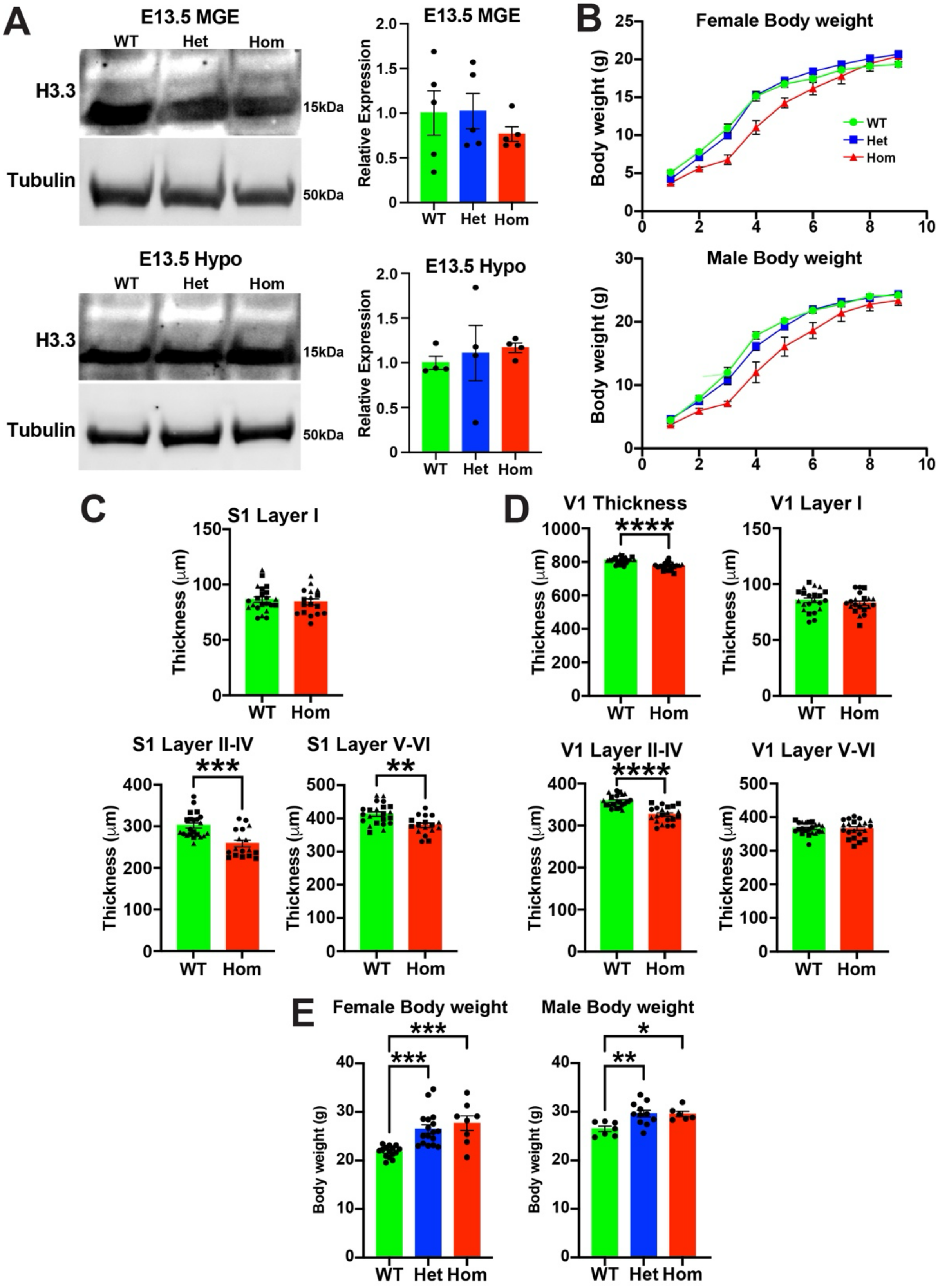

Supplementary Figure 1. Altered body weight and brain thickness in H3.3K4M Hom mice. A. Western blots of E13.5 MGE (n = 5 per genotype) and hypothalamus (n = 4 per genotype) showing H3.3 and tubulin (left), with no significant difference in total H3.3 levels (right). **B.** Weight of female (top) and male (bottom) H3.3K4M WT, Het and Hom mice from postnatal weeks 1-9. Cumulative number: 70 WT, 129 Het and 68 Hom for female mice; Cumulative number: 74 WT, 106 Het and 34 Hom for male mice. **C-D.** Decreased thickness of S1 (C) and V1 (D) cortex in Hom mice, with layers II-IV demarcated by Cux1 staining. S1: WT = 22, Hom = 17 slices, n = 3 brains per genotype. V1: WT = 22, Hom = 21 slices, n = 3 brains per genotype, sections from each brain labeled with different shape. **E.** Weight of female (left) and male (right) mice at 20 weeks. n = 15 WT females, 7 WT males, 17 Het females, 11 Het males, 8 Hom females, 6 Hom males. Data are presented as mean values +/- SEM. One-way/two-way ANOVA followed by Tukey's multiple comparison tests (A, B, E); Standard Unpaired 2-tailed t-tests (C, D): * = $p \leq .05$, ** = $p \leq .005$, *** = $p \leq .0005$, **** = $p \leq 0.0001$. Source data are provided as a Source Data file.

Supplementary Figure 2

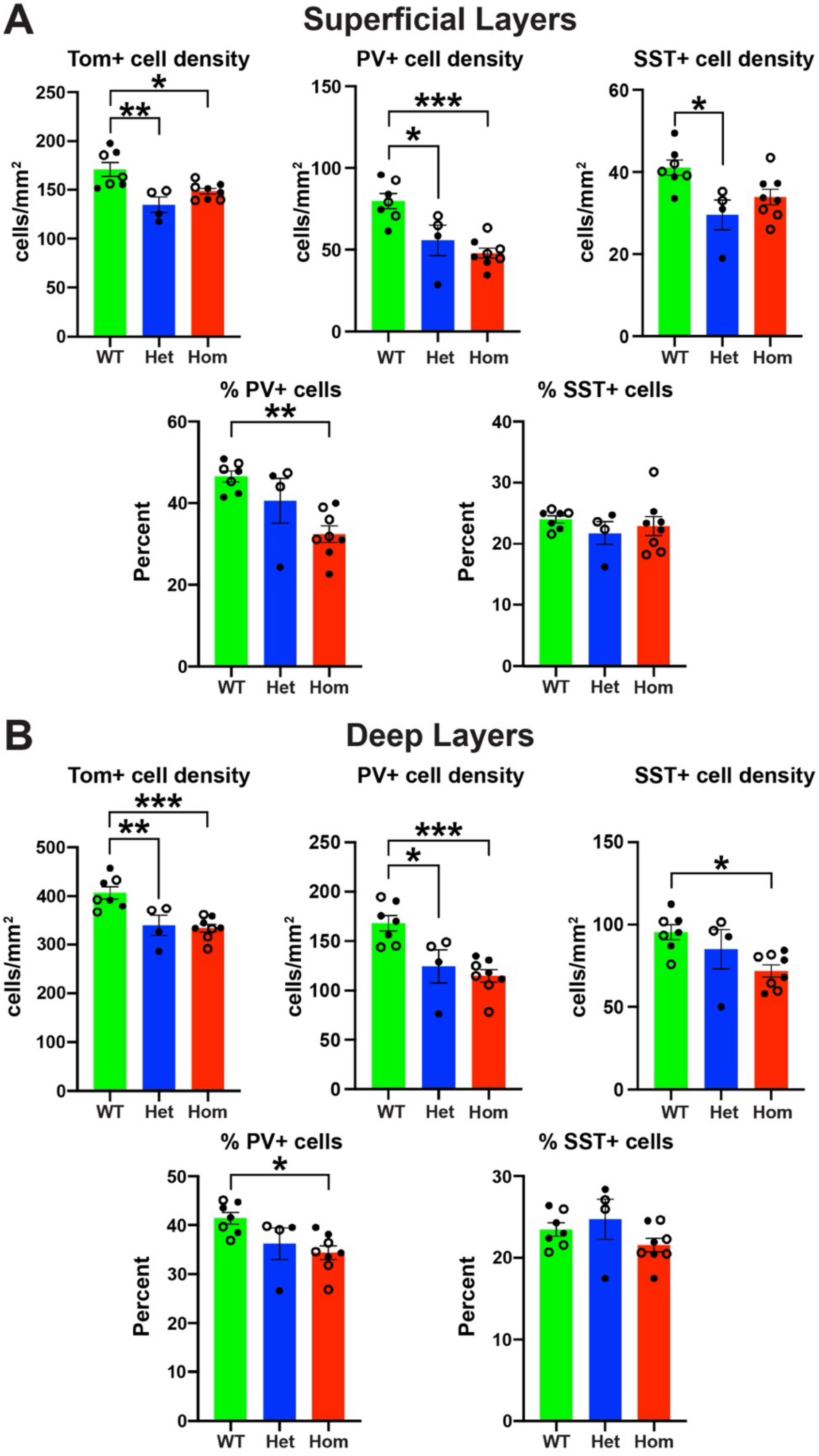

Supplementary Figure 2. Decreased MGE-derived interneurons in the superficial and deep layers of H3.3K4M Hom mice cortex. A-B. Graphs depicting the density of Tom+, PV+ and SST+ cells (top) and the percent of Tom+ cells expressing PV or SST (bottom) in the superficial layers I-III (A) and deep layers IV-VI (B) of P21 *Nkx2.1-Cre*;*H3.3K4M*;*Ai9* mice. Open circles = females, filled circles = males. n = 3 WT females, 4 WT males, 2 Het females, 2 Het males, 4 Hom females, 4 Hom males. Data are presented as mean values +/- SEM. All stats are one-way ANOVA followed by Tukey's multiple comparison tests (A, B): * = $p \leq .05$, ** = $p \leq .005$, *** = $p \leq .0005$. Source data are provided as a Source Data file.

Supplementary Figure 3

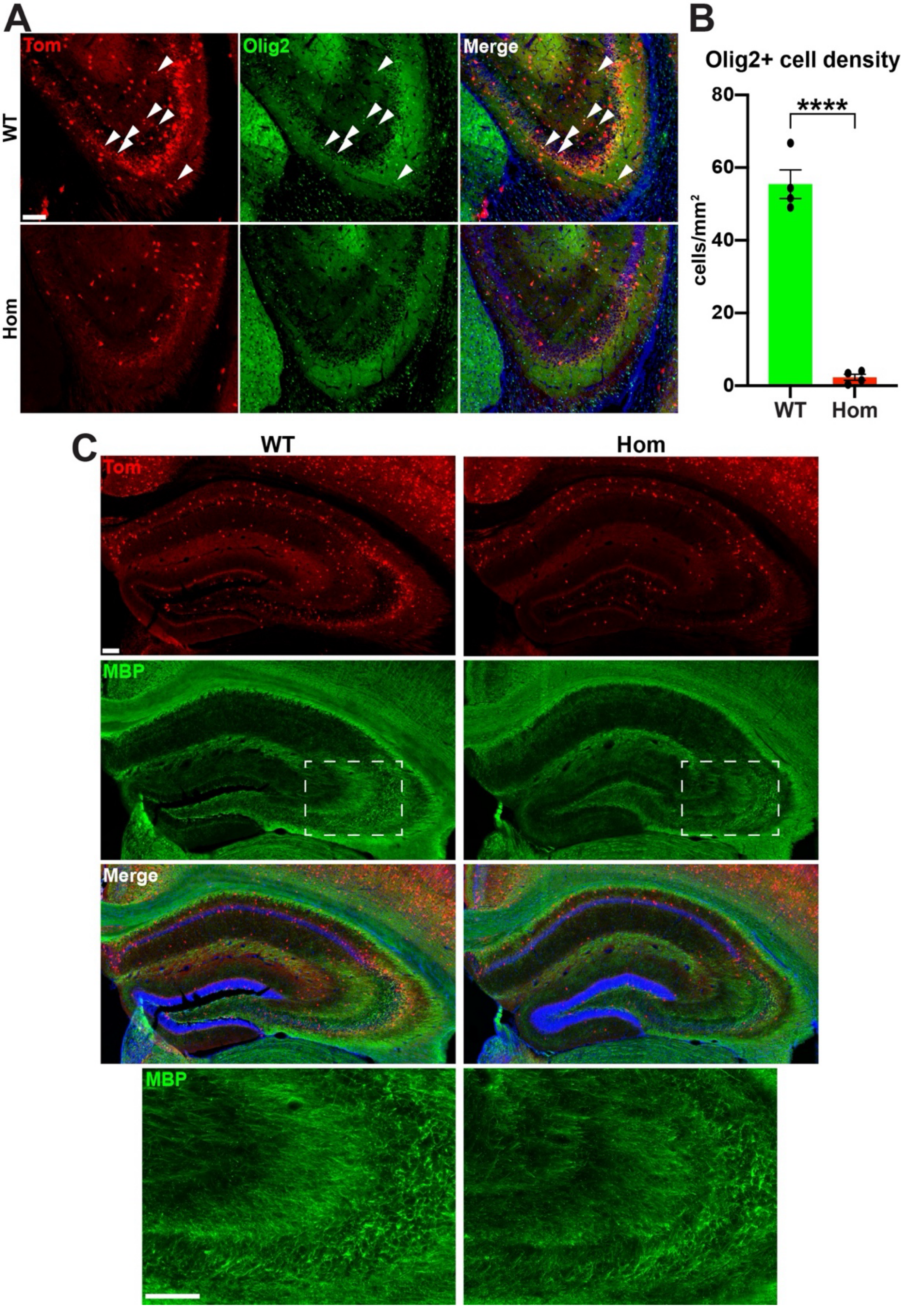

Supplementary Figure 3. Loss of MGE-derived oligodendrocytes in H3.3K4M Hom mice. A. Olig2 staining (green) through the CA3 showing loss of small cell body Tom+/Olig2+ MGE-derived oligodendrocytes (white arrowheads) in H3.3K4M Hom mice. **B.** Quantification of Tom+/Olig2+ oligodendrocytes in CA3 region of WT (n = 4) and Hom (n = 4) mice. Standard Unpaired 2-tailed t-tests: **** = $p \leq 0.0001$. **C.** Apparent reduction of myelin basic protein (MBP) in CA3 of H3.3K4M Hom mice. Data are presented as mean values +/- SEM. Scale bars = 50 μm. Source data are provided as a Source Data file.

Supplementary Figure 4

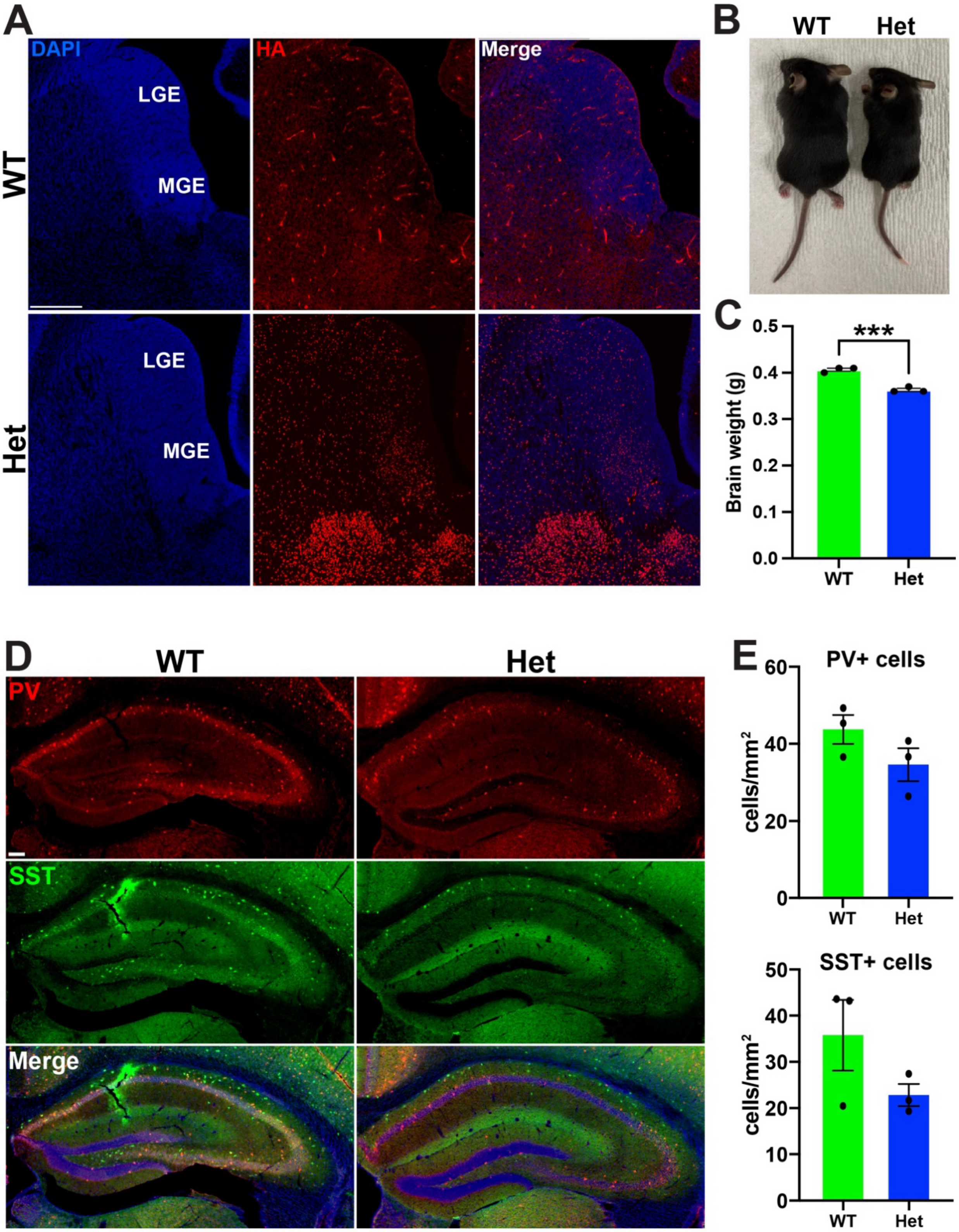

Supplementary Figure 4. Altered brain size and hippocampal interneurons in *Lhx6-Cre;H3.3K4M* Het mice. A. HA-tagged H3.3K4M expressed in MGE-derived cells in E15.5 *Lhx6-Cre;H3.3K4M* Het mice. **B-C.** *Lhx6-Cre;H3.3K4M* Het mice have reduced body weight (B) and brain weight (C) compared to WT littermates at P30. n = 3 for each genotype. **D-E.** Trend towards reduced PV+ and SST+ hippocampal interneurons in *Lhx6-Cre;H3.3K4M* Het mice at P30. n = 3 for each genotype. Scale bars = 100 μm. Data are presented as mean values +/- SEM. Standard Unpaired 2-tailed t-tests (C) and 2-tailed Welch's t-tests (E): *** = $p \leq 0.005$. Source data are provided as a Source Data file.

Supplementary Figure 5

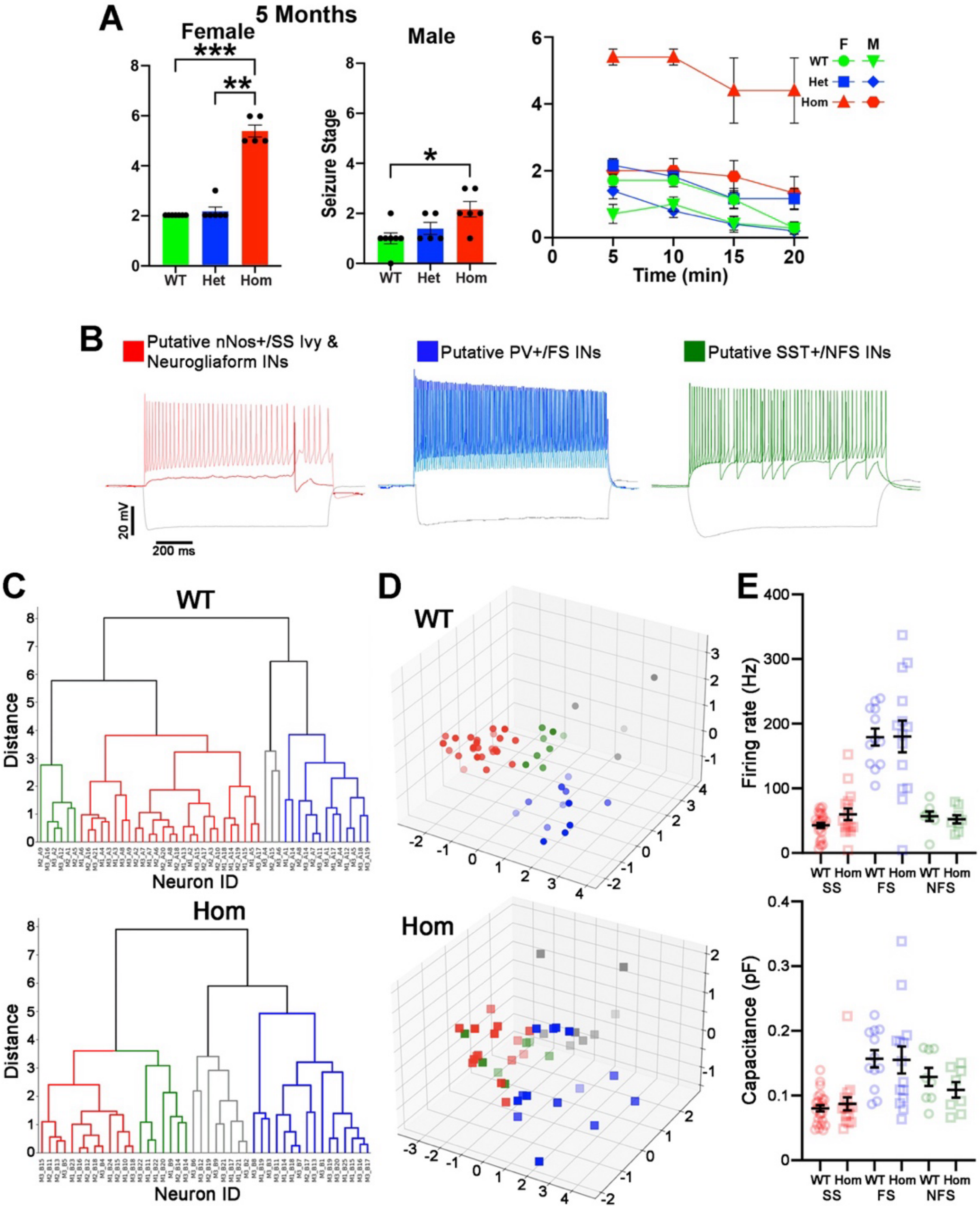

Supplementary Figure 5. Seizure scores and hippocampal interneuron intrinsic properties of WT and H3.3K4M mice. A. Maximum scores (seizure stage, left) for males and females following PTZ injection (20 mg/kg) at 5 months, and along the 20-minute observation period (right). n = 7 WT females, 7 WT males, 6 Het females, 5 Het males, 5 Hom females, 6 Hom males. **B.** Patch-clamp traces form putative nNos+/slow-spiking (SS, red), PV+/Fast Spiking (FS, blue) and SST+/non-FS (NFS, green) interneurons. **C-D.** Unbiased hierarchical clustering dendrograms (C) and PCA plots (D) of all hippocampal interneurons recorded from H3.3K4M WT (n = 49 cells) and Hom (n = 44 cells) mice. Cells that could not be classified are gray. **E.** Graphs depicting firing rate (top) and capacitance (bottom) for SS (red), FS (blue) and NFS (green) cells. Significant increase in variance in the FS population evaluated by F test in membrane capacitance (p = *) and firing rate (p = *) is observed in H3.3K4M Hom mice. Data are presented as mean values +/- SEM. Shapiro-wilk test was used to assess normality for electrophysiology data and 2-tailed unpaired t-test or 2-tailed Mann-Whitey test followed accordingly. Kruskal-Wallis followed by Dunn's multiple comparisons (A: left, middle) or two-way ANOVA followed by Tukey's multiple comparison tests (A: right) were performed: * = $p \leq .05$, ** = $p \leq .005$, *** = $p \leq .0005$. Source data are provided as a Source Data file.

Supplementary Figure 6

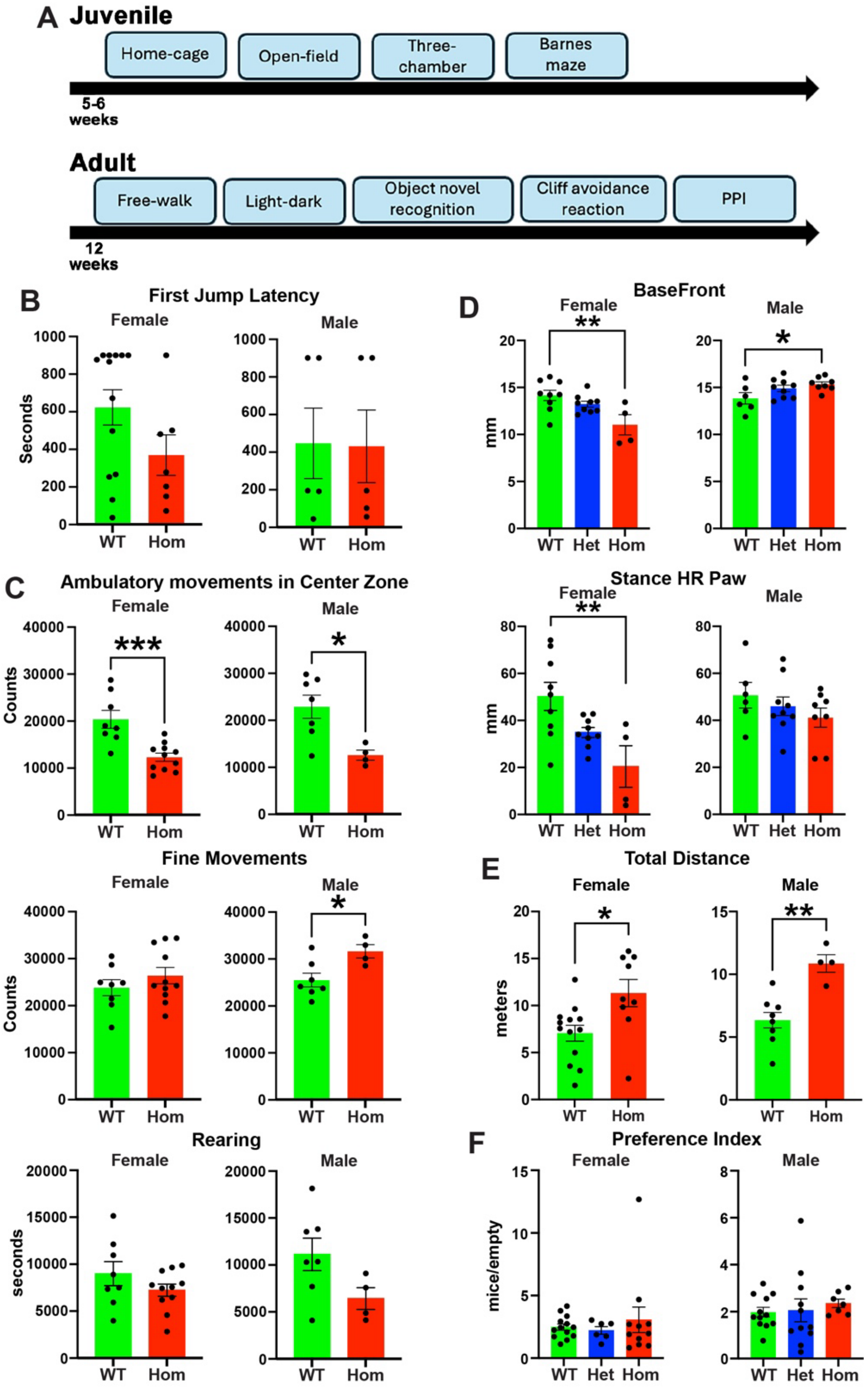

Supplementary Figure 6. Increased anxiety and impaired locomotion in H3.3K4M Hom mice. A. Timeline of behavior assays performed in juvenile and adult mice. **B.** First jump latency in the Cliff Avoidance Reaction Test. n = 13 WT females, 5 WT males, 7 Hom females, 5 Hom males. **C.** Beam break counts of ambulatory movements in the center zone (top), fine movements in the whole zone (middle) and rearing movements (bottom) over 4 days in the home cage test. n = 8 WT females, 7 WT males, 11 Hom females, 4 Hom males. **D.** BaseFront (top) and stance of hind right paw (bottom) in the free walk test. n = 9 WT females, 6 WT males, 9 Het females, 9 Het males, 4 Hom females, 8 Hom males. **E.** Total distance traveled by mice in the Barnes maze test. n = 13 WT females, 8 WT males, 9 Hom females, 4 Hom males. **F.** Preference index in the three-chamber test. n = 13 WT females, 12 WT males, 6 Het females, 11 Het males, 11 Hom females, 7 Hom males. Data are presented as mean values +/- SEM. All stats are one-way ANOVA followed by Tukey's multiple comparison tests when WT, Het and Hom mice (D, F); standard Unpaired 2-tailed t-test when only WT and Hom mice tested (B, C, E): * = $p \leq .05$, ** = $p \leq .005$, *** = $p \leq .0005$. Source data are provided as a Source Data file.

Supplementary Figure 7

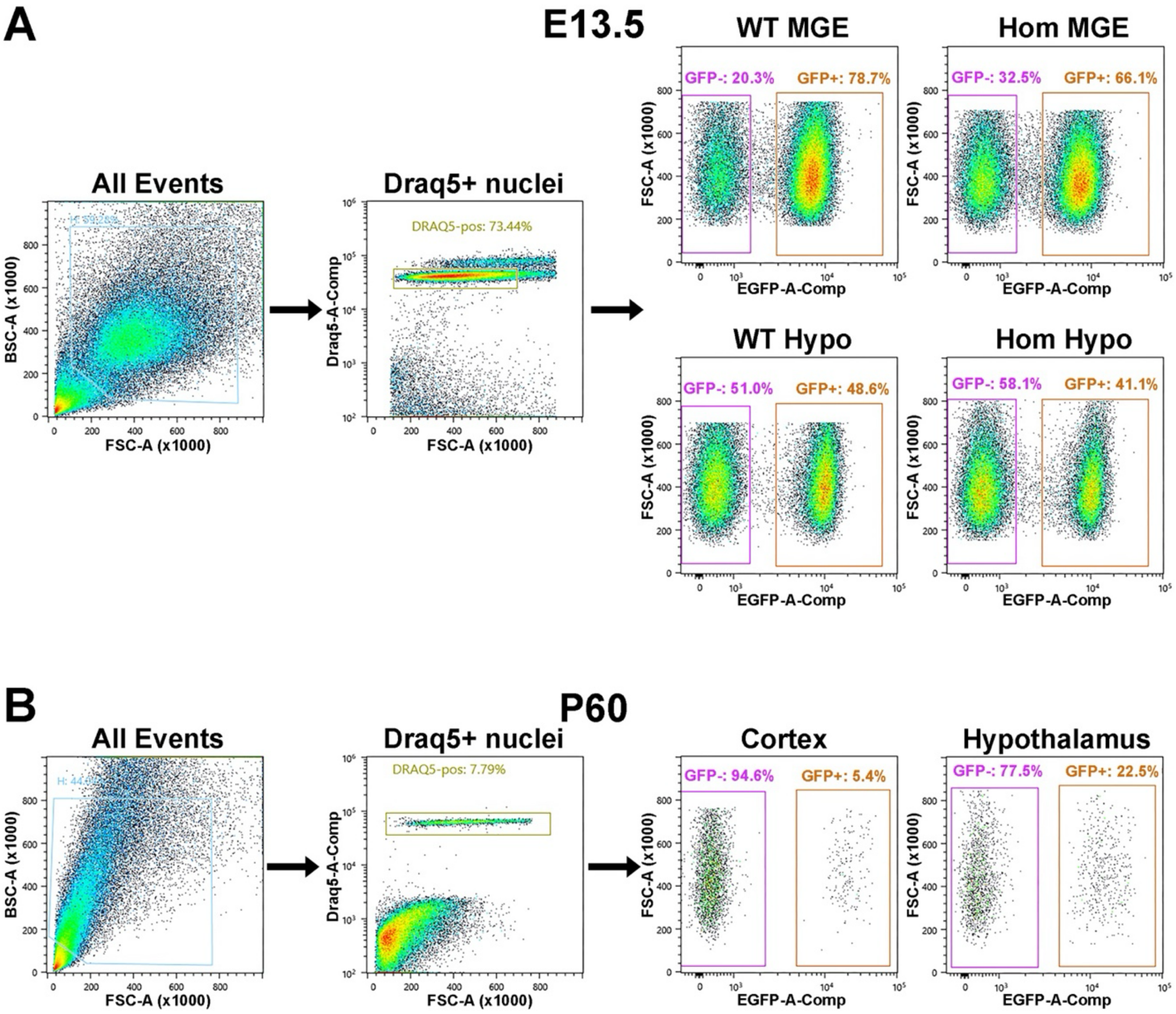

Supplementary Figure 7. Gating strategy for sorting GFP+ nuclei from embryonic and adult brains. A-B. Gating strategy for collecting GFP+ nuclei from E13.5 (A) and P60 (B) MGE/Cortex and hypothalamus. Putative nuclei are gated by forward scatter (FSC) vs. back/side scatter (SSC), then gated for the nuclear label Draq5 by Draq-5 vs. FSC, then GFP+ nuclei were collected on the EGFP-A vs. FSC-A gate. Lower percentages of GFP+ nuclei/Draq5+ nuclei were consistently obtained from E13.5 MGE and hypothalamus in Hom mice compared to WT; this was not observed in P60 mice.

Supplementary Figure 8

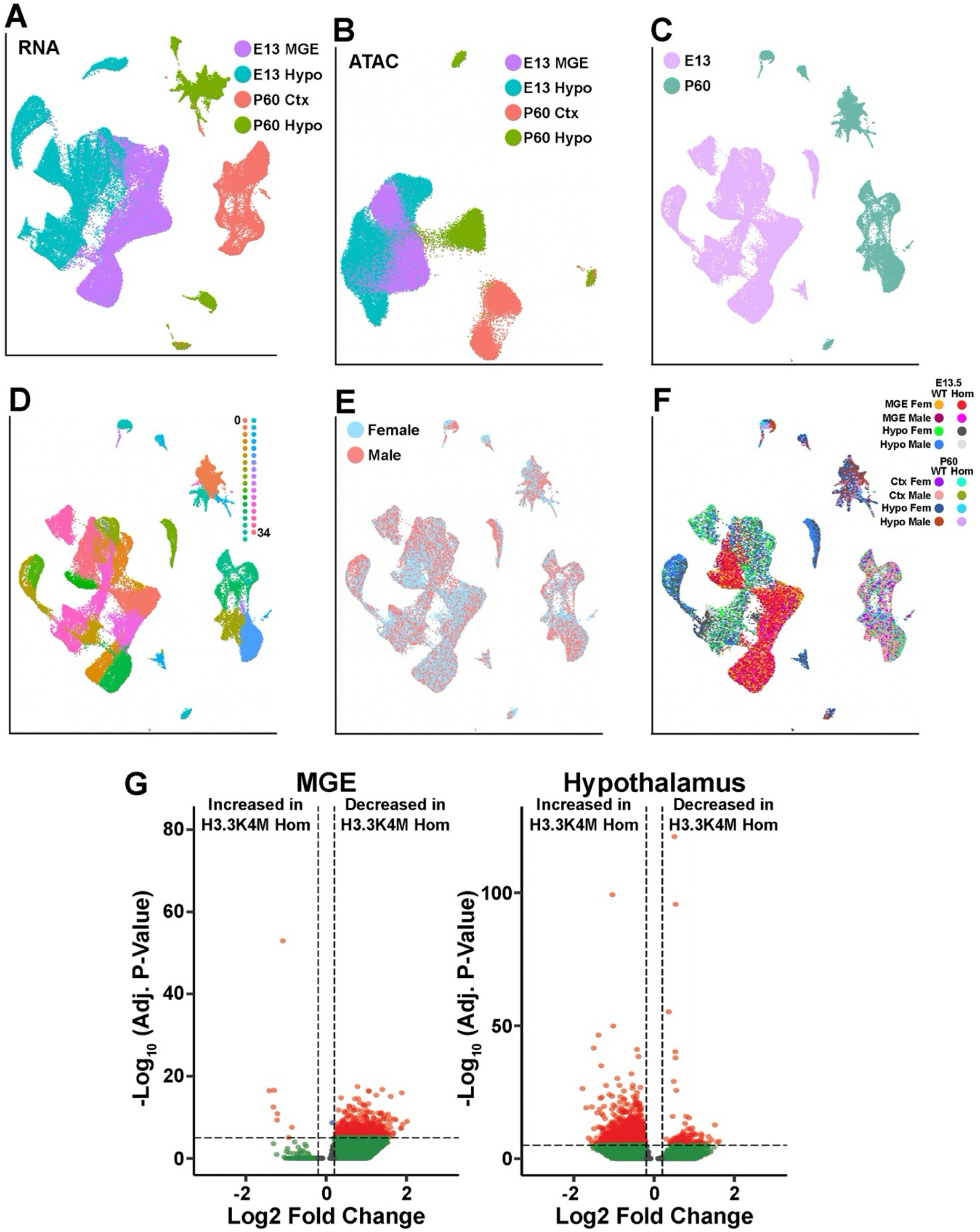

Supplementary Figure 8. Total integrated Multiome-seq (snRNA-seq and snATAC-seq) data of 16 samples. All data is combined male & female samples. **A-B.** RNA-only (A) and ATAC-only (B) UMAP plots of E13.5 MGE, E13.5 hypothalamus, P60 cortex and P60 hypothalamus annotated by age and tissue. **C-F.** Integrated UMAP plots of E13.5 MGE, E13.5 hypothalamus, P60 cortex and P60 hypothalamus annotated by age (C), putative cell clusters (D), sex (E) and library id (F). **G.** Volcano plots depicting differentially accessible peaks in embryonic MGE (left) and hypothalamus (right). Two-sided Wilcoxon rank sum test with Bonferroni correction (G) was used. Source data are provided as a Source Data file.

Supplementary Figure 9

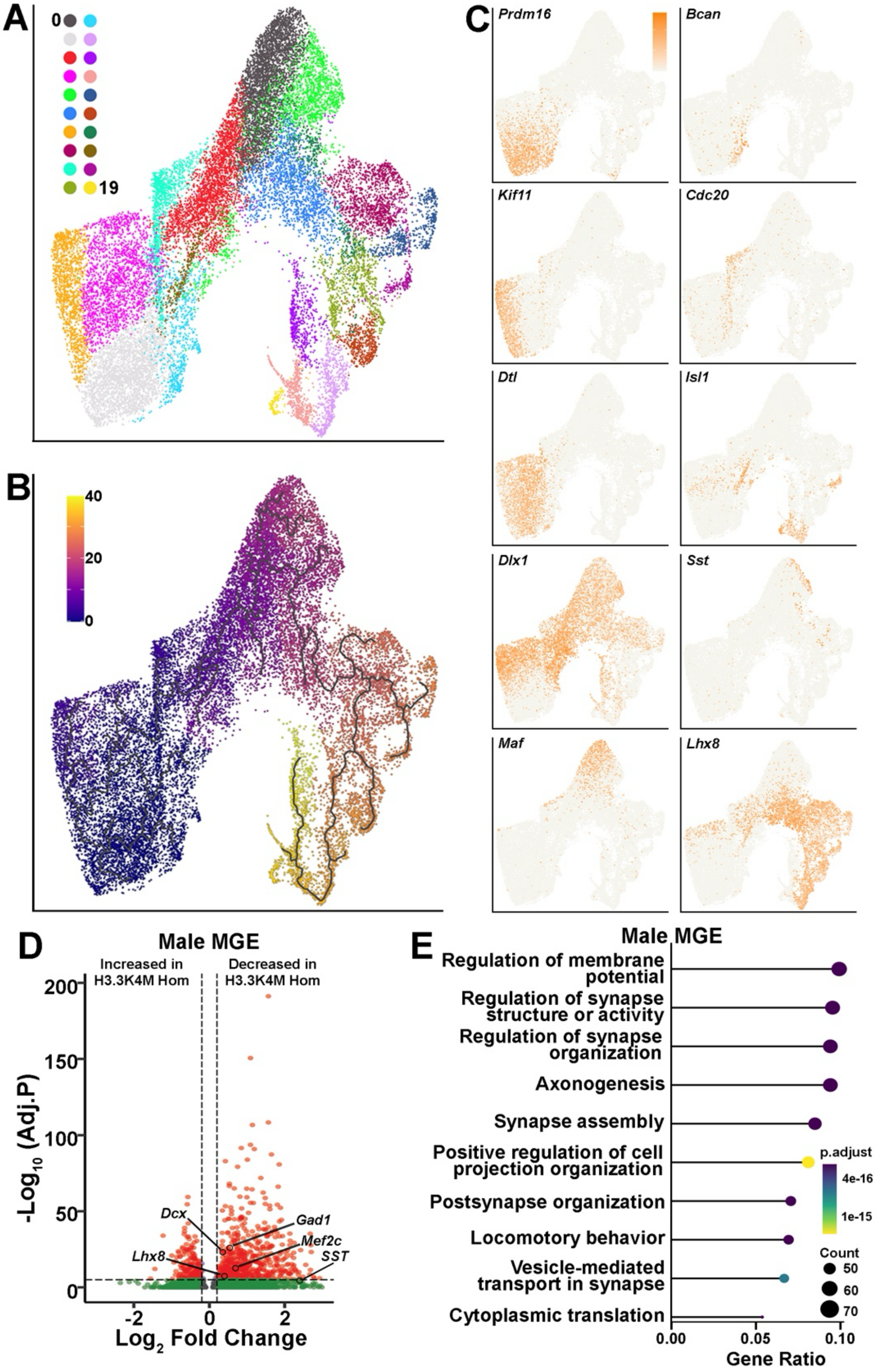

Supplementary Figure 9. Single cell sequencing of E13.5 MGE. Data is combined male & female samples unless stated otherwise. **A-C.** Integrated UMAP plots of E13.5 MGE annotated by Seurat clusters (A), pseudotime developmental trajectory (B) and specific marker genes (C). **D.** Volcano plot depicting DEGs in male E13.5 H3.3K4M MGE. **E.** clusterProfiler GO enrichment top biological processes for DEGs of male E13.5 MGE. Two-sided Wilcoxon rank sum test with Bonferroni correction (D) and one-sided Fisher's exact with BH correction (E) were used. Source data are provided as a Source Data file.

Supplementary Figure 10

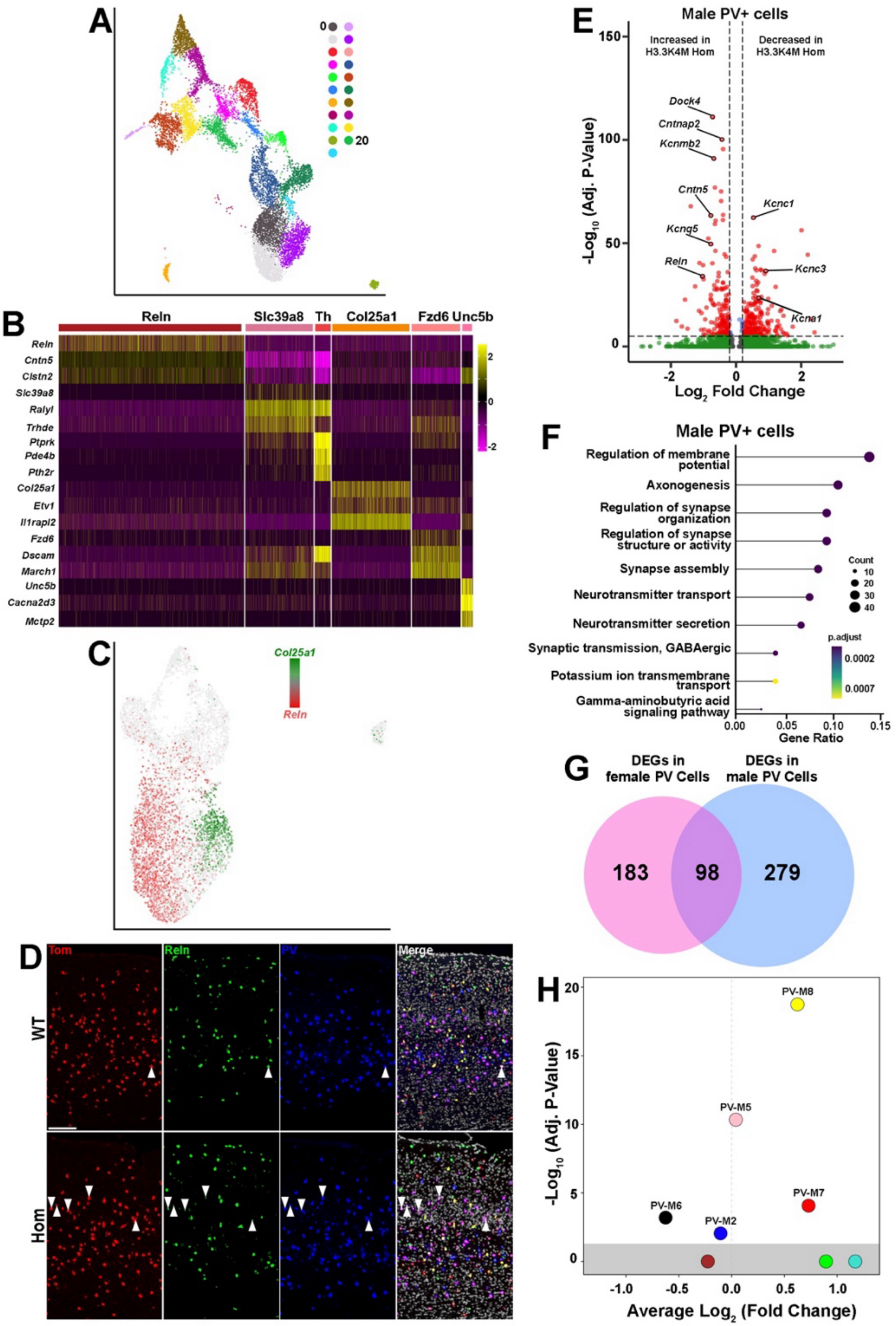

Supplementary Figure 10. Single cell sequencing of P60 cortical PV+ cells. Data is combined male & female samples unless stated otherwise. **A.** Integrated UMAP plots of MGE-derived interneurons in P60 cortex annotated by Seurat clusters. **B.** Heatmap depicting genes enriched in PV+ subtypes. **C.** RNA-only UMAP plot of PV+ interneurons depicting *Reln* and *Col25a1* expression. **D.** P60 cortex showing increased number of Tom+/PV+/Reln+ cortical interneurons (white arrowheads) in H3.3K4M Hom mice. Scale bar = 100 μm. **E.** Volcano plot depicting DEGs in male P60 H3.3K4M Hom PV+ cells. **F.** clusterProfiler GO enrichment top biological processes for DEGs of male P60 PV+ cells. **G.** Venn diagram comparing DEGs in PV+ cells between WT and Hom mice divided by sex. **H.** Volcano plot depicting module eigengene differences identified by co-expression analysis in PV+ population between H3.3K4M WT and Hom mice. Two-sided Wilcoxon rank sum test with Bonferroni correction (E) and one-sided Fisher's exact with BH correction (F) were used. Source data are provided as a Source Data file.

Supplementary Figure 11

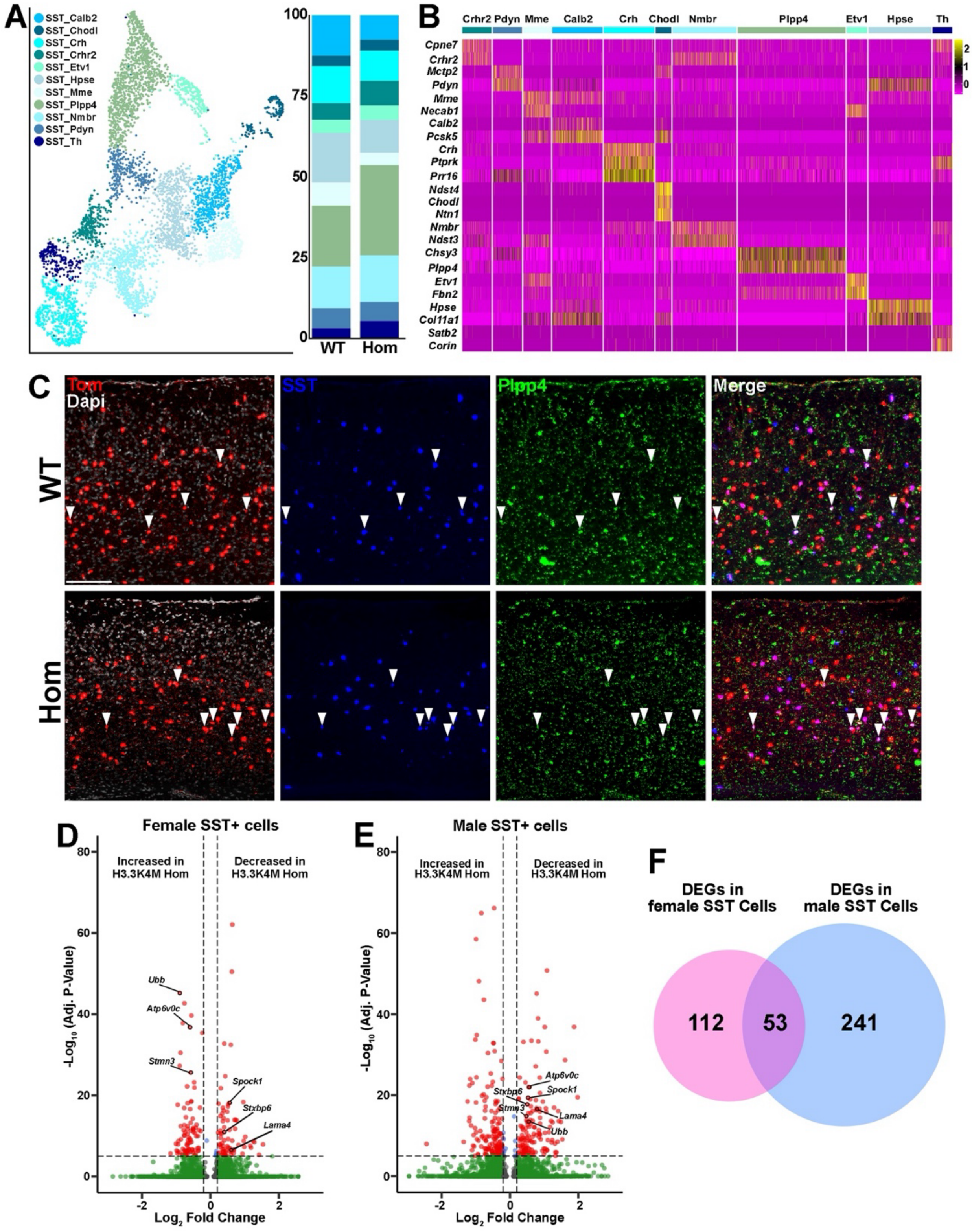

Supplementary Figure 11: Single cell sequencing of P60 cortical SST+ cells. Data is combined male & female samples unless stated otherwise. **A.** RNA-only UMAP plot of SST+ cells annotated by 11 SST+ subtypes (left), and relative proportions of SST+ subtypes in H3.3K4M WT and Hom cortices (right). **B.** Heatmap depicting genes enriched in SST+ subtypes. **C.** P60 cortex showing increased proportion of Tom+/SST+/Plpp4+ cortical interneurons (white arrowheads) in H3.3K4M Hom mice. Scale bar = 100 μm. **D-E.** Volcano plots depicting DEGs in female P60 H3.3K4M Hom SST+ cells in female (D) and male (E) mice. **F.** Venn diagram comparing DEGs in PV+ cells between WT and Hom mice divided by sex. Two-sided Wilcoxon rank sum test with Bonferroni correction (D, E) was used. Source data are provided as a Source Data file.

Supplementary Figure 12

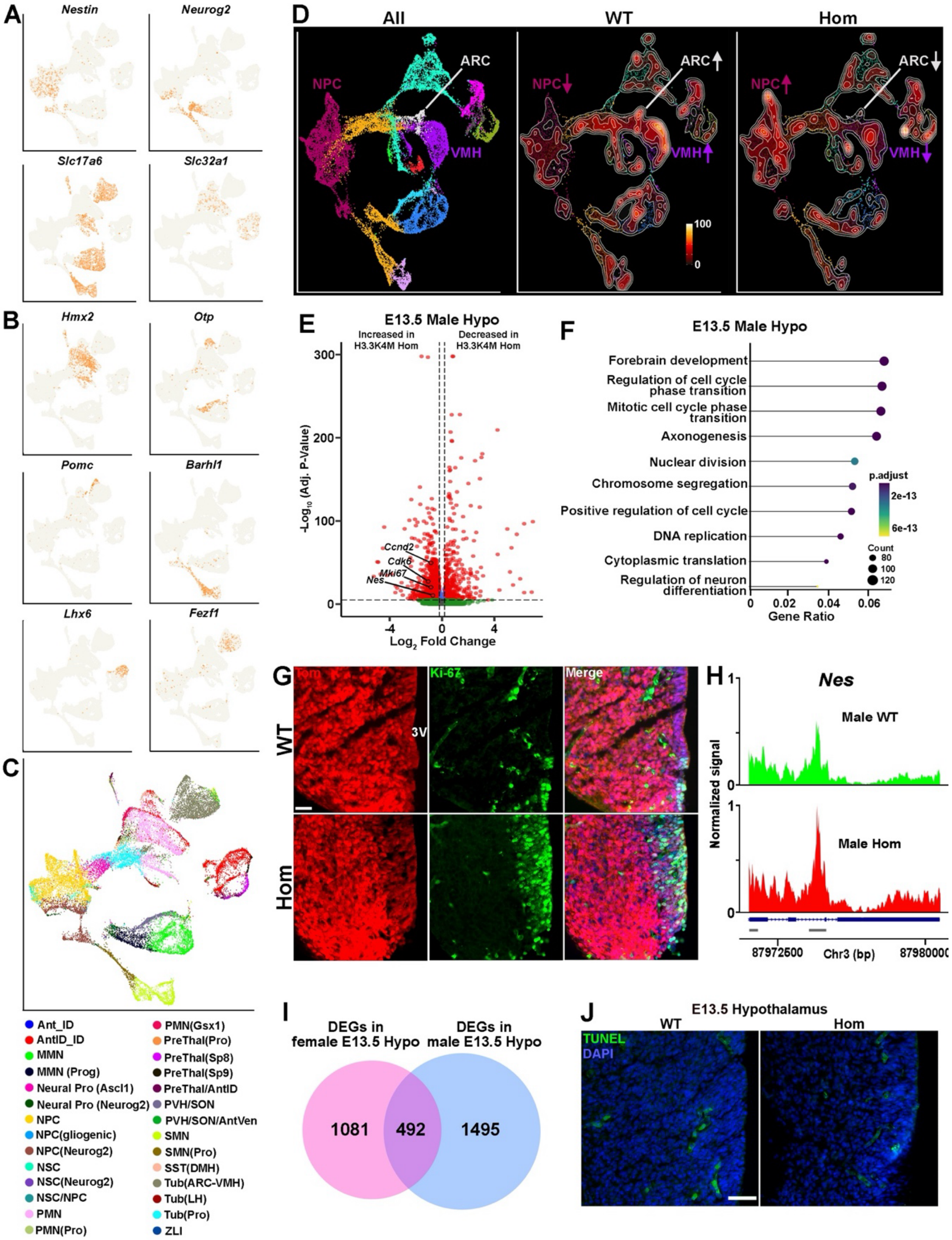

Supplementary Figure 12. Transcriptome changes in numerous hypothalamic cell types in H3.3K4M Hom mice. Data is combined male & female samples unless stated otherwise. **A.** Markers for apical progenitors (*Nestin*), neuronal precursors (*Neurog2*), postmitotic glutamatergic neurons (*Slc17a6*) and postmitotic GABAergic neurons (*Slc32a1*). **B.** Markers of region-specific hypothalamic nuclei PMN (*Hmx2*), DMH and PVN/SON (*Otp*), ID & TT (*Lhx6*), ARC (*Pomc*), SMN (*Barhl1*) and VMH (*Fezf1*). **C.** Integrated UMAP plot of Nkx2.1-lineage cells from E13.5 hypothalamus with detailed annotation of hypothalamic nuclei. **D.** RNA-only UMAP plots annotated by hypothalamic nuclei (left, color coding same as Fig 8A), and WT and Hom UMAPs separated and overlayed with heatmaps highlighting cell densities for each genotype (right). **E.** Volcano plot depicting DEGs in male E13.5 H3.3K4M Hom hypothalamus. **F.** clusterProfiler GO enrichment top biological process for DEGs of male E13.5 hypothalamus. **G.** E13.5 hypothalamus from H3.3K4M WT and Hom mice stained for Ki67 (green), DAPI (blue), with Tom+ Nkx2.1-linage cells. 3V = Third ventricle. Scale bar = 100 µm. **H.** Tracks showing increased genomic accessibility at *Nes* promoter and intronic enhancer (gray bars) in male E13.5 H3.3K4M Hom hypothalamus. **I.** Venn diagram comparing DEGs in E13.5 hypothalamus between WT and Hom mice divided by sex. **J.** E13.5 hypothalamus from H3.3K4M WT and mice with TUNEL signal (green) and DAPI (blue). Scale bar = 100 µm. Two-sided Wilcoxon rank sum test with Bonferroni correction (E) and one-sided Fisher's exact with BH correction (F) were used. Source data are provided as a Source Data file.

Supplementary Figure 13

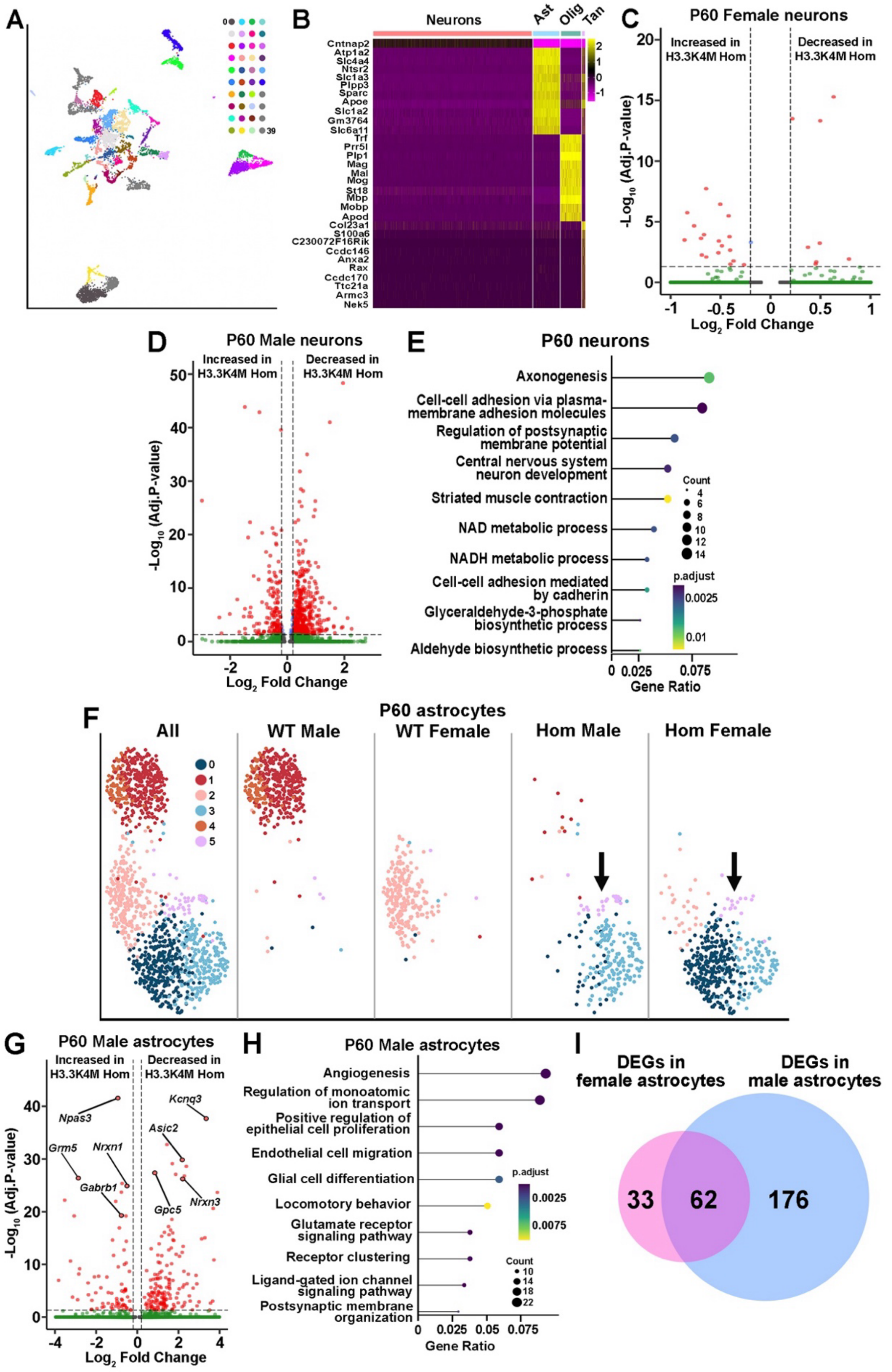

Supplementary Figure 13. Transcriptome changes in P60 hypothalamic cells in H3.3K4M Hom mice. Data is combined male & female samples unless stated otherwise. **A.** Integrated UMAP plot of P60 hypothalamus annotated by Seurat clusters. **B.** Heatmap depicting critical genes that define astrocytes, oligodendrocytes and tanycytes. **C-D.** Volcano plot depicting DEGs of female (C) and male (D) P60 hypothalamic neurons. **E.** clusterProfiler GO enrichment of biological process for DEGs of combined male and female P60 hypothalamic neurons. **F.** RNA-only UMAP plots of astrocytes annotated by putative astrocyte subtypes, with all samples combined (left) and separated by sex and genotype. **G.** Volcano plot depicting DEGs of male P60 hypothalamic astrocytes. **H.** clusterProfiler GO enrichment items of biological process for DEGs in male P60 astrocytes. **I.** Venn diagram comparing DEGs in P60 hypothalamic astrocytes between WT and Hom mice divided by sex. Two-sided Wilcoxon rank sum test with Bonferroni correction (C, D, G) and one-sided Fisher's exact with BH correction (E, H) were used. Source data are provided as a Source Data file.

Supplementary Figure 14

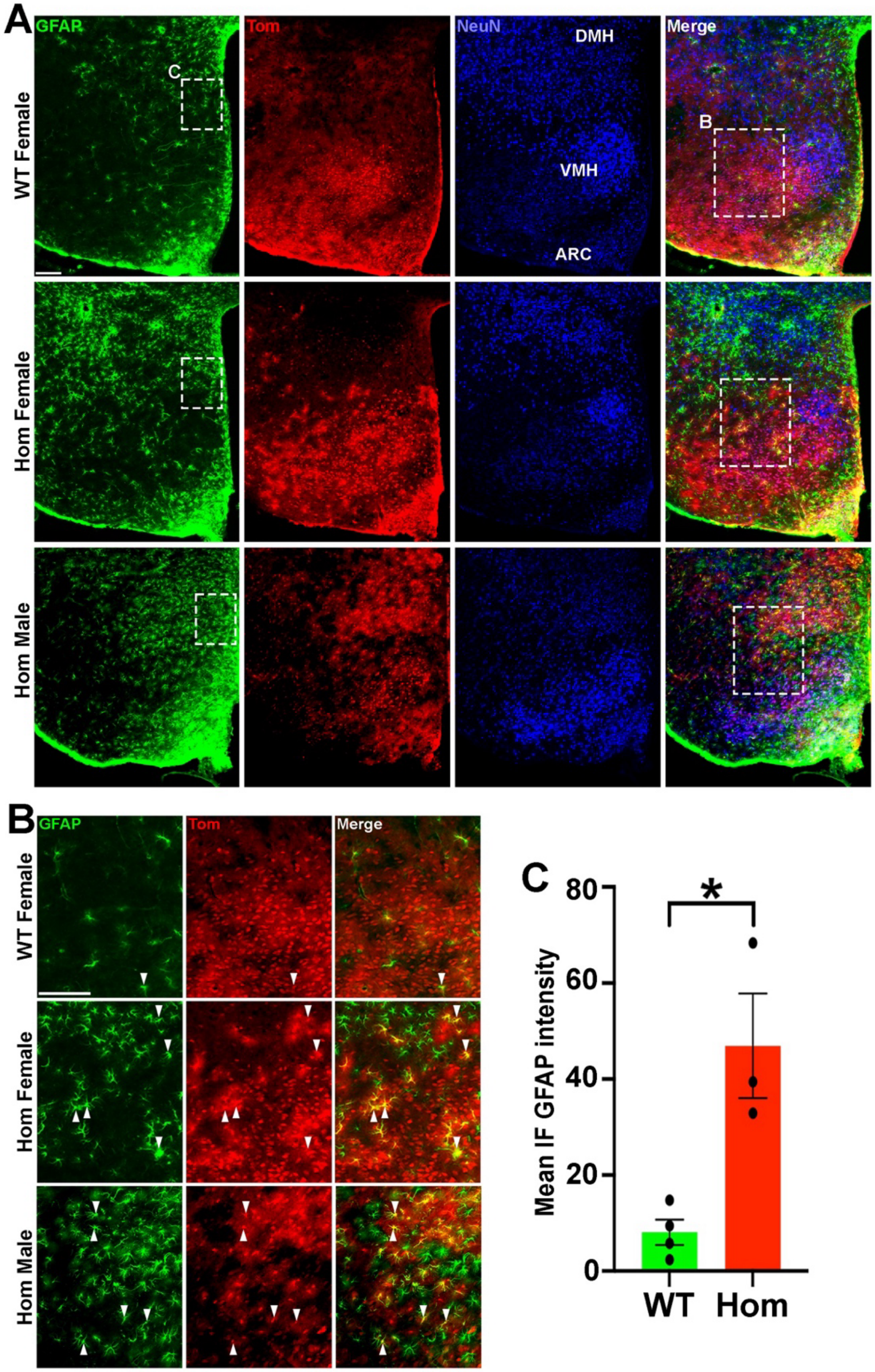

Supplementary Figure 14. Dysregulation of glia cells in adult hypothalamus of H3.3K4M Hom mice. A-C. Adult hypothalamus from H3.3K4M WT and Hom male and female mice stained for GFAP (green), NeuN (blue), with Nkx2.1-linage cells expressing tdTomato. Scale bar = 100 µm. White rectangles in A indicate higher magnification regions (B), and regions used to measure immunofluorescence (IF) intensity of GFAP signal (C). n = 4 WT and 3 Hom mice. Data are presented as mean values +/- SEM. Standard Unpaired 2-tailed t-tests: * = $p \leq 0.05$. ARC, Arcuate nucleus; VMH, ventromedial hypothalamus; DMH, dorsomedial hypothalamus. Source data are provided as a Source Data file.
