# Supplementary material for: Reducing methylation of histone 3.3 lysine 4 in the medial ganglionic eminence and hypothalamus recapitulates neurodevelopmental disorder phenotypes
Source: Nat Commun. 2026 Feb 20;17:2984. doi: 10.1038/s41467-026-69248-9 (PMC13035845; doi:10.1038/s41467-026-69248-9)
Supplement: Supplementary file 2 — Description of Additional Supplementary Files [file 41467_2026_69248_MOESM2_ESM.pdf]

Description of Additional Supplementary Files

File name: Supplementary Video 1
Description: Video of P60 H3K4 Hom mouse displaying spontaneous seizures, in cage with WT and Het littermates.

File name: Supplementary Data 1
Description: Table displaying number of cells sequenced for each single cell Multiome sequencing reaction, with average reads and gene/fragments per cell.

File name: Supplementary Data 2
Description: Spreadsheet contains n values, cell counts & other quantitative analyses, detailed statistics, and images of unedited Western Blots for all relevant experiments in the manuscript.

File name: Supplementary Data 3
Description: Spreadsheet contains log fold change and adjusted p-values used to determine differentially expressed genes for comparisons of all single cell sequencing experiments described in the manuscript.
