# Supplementary material for: Reducing methylation of histone 3.3 lysine 4 in the medial ganglionic eminence and hypothalamus recapitulates neurodevelopmental disorder phenotypes
Source: Nat Commun. 2026 Feb 20;17:2984. doi: 10.1038/s41467-026-69248-9 (PMC13035845; doi:10.1038/s41467-026-69248-9)
Supplement: Supplementary file 7 — Reporting Summary [file 41467_2026_69248_MOESM7_ESM.pdf]

nature portfolio

Corresponding author(s): Timothy J. Petros

Last updated by author(s): Jan 12, 2026

Reporting Summary

Nature Portfolio wishes to improve the reproducibility of the work that we publish. This form provides structure for consistency and transparency in reporting. For further information on Nature Portfolio policies, see our Editorial Policies and the Editorial Policy Checklist.

Statistics

For all statistical analyses, confirm that the following items are present in the figure legend, table legend, main text, or Methods section.

n/a	Confirmed	
☐	☒	The exact sample size (*n*) for each experimental group/condition, given as a discrete number and unit of measurement
☐	☒	A statement on whether measurements were taken from distinct samples or whether the same sample was measured repeatedly
☐	☒	The statistical test(s) used AND whether they are one- or two-sided<br>*Only common tests should be described solely by name; describe more complex techniques in the Methods section.*
☐	☒	A description of all covariates tested
☐	☒	A description of any assumptions or corrections, such as tests of normality and adjustment for multiple comparisons
☐	☒	A full description of the statistical parameters including central tendency (e.g. means) or other basic estimates (e.g. regression coefficient) AND variation (e.g. standard deviation) or associated estimates of uncertainty (e.g. confidence intervals)
☐	☒	For null hypothesis testing, the test statistic (e.g. *F*, *t*, *r*) with confidence intervals, effect sizes, degrees of freedom and *P* value noted<br>*Give P values as exact values whenever suitable.*
☒	☐	For Bayesian analysis, information on the choice of priors and Markov chain Monte Carlo settings
☒	☐	For hierarchical and complex designs, identification of the appropriate level for tests and full reporting of outcomes
☒	☐	Estimates of effect sizes (e.g. Cohen's *d*, Pearson's *r*), indicating how they were calculated

Our web collection on statistics for biologists contains articles on many of the points above.

Software and code

Policy information about availability of computer code

Data collection	Cells were sequenced with paired-end (50 x 50 bp) using an Illumina HiSeq 2500 or NovaSeq 6000. All images were captured on an Olympus VS200 scanner (VS200 ASW) or Zeiss Axioimager.M2 (with Zen Blue software). Data collection for behavioral tests used video tracking system ANY-maze (Stoelting Company), Photobeam Activity System-Home Cage (PAS-HC, SD Instruments), FreeWalkScan 2.0 (CleverSys Inc.) and SR-LAB-Startle Response System (San Diego Instruments). Body composition was collected via EchoMRI100 analyzer (Echo Medical Systems). Blots were visualized with ChemiDoC MP Imaging System (Bio-Rad). Electrophysiology experiments of recordings were performed using a Multiclamp 700B amplifier and acquired using pCLAMP 10,4 software (Molecular Devices).
Data analysis	Softwares or packages used for analysis: Cell Ranger ARC (v2.0.0), Seurat (v5.2.1), R (v4.4.2, https://cran.r-project.org), RStudio (v2024.09.0 +375), scDblFinder (v1.20.0), Signac (v1.14.0), EnhancedVolcano (v1.24.0, https://github.com/kevinblighe/EnhancedVolcano), clusterProfiler (v4.14.0), org.Mm.eg.db (v3.20.0), hdWGCNA (v0.4.03), Monocle 3 (v1.3.7), EnsDb.Mmusculus.v79 (v2.99.0), patchwork (v1.3.0), presto (v1.0.0), ggplot2 (v3.5.1), GenomicRanges (v1.57.2), here (v1.0.1), SingleCellExperiment (v1.28.0), BiocParallel (v1.39.0), dplyr (1.1.4), Adobe Photoshop (v24.7.5), ImageJ2 (V2.9.0), Microsoft Excel (v16.96.1), Prism (v10.6.1), FreewalkScanTM (v2.0) and ANY-maze (Stoelting Company). Analysis of whole cell patch clamp recordings was performed using the AutoAnt Software. Frequency variance data was obtained from the power spectra described above using the Axograph software (Kagi, Berkeley, CA, USA).

For manuscripts utilizing custom algorithms or software that are central to the research but not yet described in published literature, software must be made available to editors and reviewers. We strongly encourage code deposition in a community repository (e.g. GitHub). See the Nature Portfolio guidelines for submitting code & software for further information.

Data

Policy information about availability of data

All manuscripts must include a data availability statement. This statement should provide the following information, where applicable:

- Accession codes, unique identifiers, or web links for publicly available datasets
- A description of any restrictions on data availability
- For clinical datasets or third party data, please ensure that the statement adheres to our policy

All single nuclei Multiome reactions (snRNA-seq and snATAC-seq) reported here have been deposited in GEO under accession numbers GSE293751 (scRNA-Seq), GSE293655 (snATAC-Seq) and GSE293881 (combined snRNA & snATAC datasets), and are publicly accessible . Our computational pipeline to analyze single nuclei Multiome data (called 'multiome-wf') is publicly available at: https://github.com/NICHD-BSPC/multiome-wf/, with documentation located at: https://nichd-bspc.github.io/multiome-wf/. This pipeline was used for some analysis in this manuscript, while other times data was analyzed using other packages described in the 'Software & Code' section above and detailed in the manuscript. Source data files have been uploaded, and additional data & analyses described in the manuscript has been uploaded to Synapse.org (https://www.synapse.org/Synapse:syn72376922/wiki/638833).

Research involving human participants, their data, or biological material

Policy information about studies with human participants or human data. See also policy information about sex, gender (identity/presentation), and sexual orientation and race, ethnicity and racism.

Reporting on sex and gender	No humans or human tissue was used in this study.
Reporting on race, ethnicity, or other socially relevant groupings	N/A
Population characteristics	N/A
Recruitment	N/A
Ethics oversight	N/A

Note that full information on the approval of the study protocol must also be provided in the manuscript.

Field-specific reporting

Please select the one below that is the best fit for your research. If you are not sure, read the appropriate sections before making your selection.

[x] Life sciences [ ] Behavioural & social sciences [ ] Ecological, evolutionary & environmental sciences

For a reference copy of the document with all sections, see nature.com/documents/nr-reporting-summary-flat.pdf

Life sciences study design

All studies must disclose on these points even when the disclosure is negative.

Sample size

No sample-size calculation was performed prior to experimentation. As 25% of homozygous mice die by 4 weeks and 50% die by 20 weeks, this limits the number of Hom animals available for some assays.

For single cell Mulitome sequencing experiments, we chose to perform 2 replicates for each brain region, genotype and age (8 different conditions, 16 total samples, see Supp Table 1), with each replicate containing 1 male and 1 female sample. Many high quality single cell sequencing studies only perform n=1 for each sample; here we performed 2 replicates for each experiment, and having separate male/female replicates allowed us to both increase cell number for each tissue/age/genotype, as well as gain important insights about sex differences.

For slice whole-cell patch recordings, we used 3 WT and 3 Hom mice with the goal of obtaining 40+ cells from each (n = 49 WT cells and 44 Hom cells). We predicted these cell numbers were sufficient to identify different interneuron subclasses in the hippocampus and related analysis based on previous work from the McBain lab.

For gamma oscillation/network analysis ex vivo slice analysis, we wanted a minimum of 4 WT and Hom brains, with goal of obtaining recordings from 6-10 slices/brain (WT = 4 brains, 39 slices; Hom = 5 brains, 41 slices). Previous work from the McBain lab suggested these n's would be sufficient to identify any significant differences.

For interneuron cell counts, we wanted a minimum of 5 different WT and Hom brains, with cell counts performed on 3-4 non-consecutive sections per brain in the cortex and hippocampus. This resulted in > 300 Tom+ cortical cells & >100 Tom+ cortical cells per WT brain, which the Petros lab has previously determined as sufficient to identify the 2-3 distinct MGE-derived cell types in the brain, and identify significant differences between genotypes (See Data Sheet 1).

For all behavior assays, the number of mice for each group (genotype & sex) ranged from 4-14. Averaging all groups (genotype+sex) for all behavior assays results in an average of 8.5 mice for each group in all behavior assays in presented in Figs 6, 7 & Extended Data Fig 6. We believe this average of 8.5 mice for each sex/genotype/assay is higher than most publications and is sufficient to identify significant changes

between genotypes and sexes for each assay.

After learning that 50-80% of Hom mice died after PTZ injection, we sought a minimum of 4-5 mice of each sex at each age (range from 4-7 mice per age/sex/genotype). This minimized the number of mice that did not recover from the PTZ injection while also providing a minimum n to obtain significant results.

Data exclusions

1 WT mouse was excluded from the e-phys gamma oscillation/network analysis dataset (Fig. 4D-J) because it never generated gamma oscillations due to technical issues that were discovered after the recording session. Thus only 4 WT mice were included here in comparison to 5 Hom mice. No other data were exluded from this study.

Replication

As detailed in the 'Sample size' section above, we believe the n's for each experiment provided sufficient replication, and the detailed description in the Methods section of the manuscript should increase the likelihood that our findings can be reproduced by other groups.

Randomization

The randomization does not apply as there were no group allocation before experiments. For behavioral tests, mice were randomly assigned to experimental series in the tested day. Group allocation of genotype and sex were determined after data collection and analysis.

Blinding

All cell counts, behavior assays and electrophysiology experiments were performed blind to genotype.

Reporting for specific materials, systems and methods

We require information from authors about some types of materials, experimental systems and methods used in many studies. Here, indicate whether each material, system or method listed is relevant to your study. If you are not sure if a list item applies to your research, read the appropriate section before selecting a response.

Materials & experimental systems

n/a	Involved in the study	
☐	☒	Antibodies
☒	☐	Eukaryotic cell lines
☒	☐	Palaeontology and archaeology
☐	☒	Animals and other organisms
☒	☐	Clinical data
☒	☐	Dual use research of concern
☒	☐	Plants

Methods

n/a	Involved in the study	
☒	☐	ChIP-seq
☐	☒	Flow cytometry
☒	☐	MRI-based neuroimaging

Antibodies

Antibodies used

rat anti-SST (1:300) Millipore MAB354
goat anti-PV (1:1000) Swant PVG213
rabbit anti-nNos (1:500) Millipore MAB5380
rabbit anti-Olig2 (1:500) Sigma-Aldrich AB9610
rat anti-HA (1:200) Roche 11867423001
mouse anti-GFAP (1:500) Sigma Aldrich G3893
rabbit anti-NeuN (1:500) abcam ab177487
rabbit anti-Vimentin (1:500) Cell Signaling Technology 5741T
mouse anti-MBP (1:1000) Invitrogen MA1-10837
rabbit anti-H3K4me3 (1:500) Cell Signaling Technology 9751S
rabbit anti-H3K4me1 (1:500) abcam ab8895
rabbit anti-H3K4me2 (1:500) abcam ab7766
mouse anti-Ki67 (1:200) BD biosciences 550609
rabbit anti Phospho-Histone H3 (Ser10) (1:500) ThermoFisher PA5-17869
rabbit anti-Cux1 (1:300) Proteintech 11733-1-AP
humanFAB rhodamine anti-Tubulin (1:2000) BioRad 12004166 (Western blot only)
rabbit anti-H3.3 (1:1000) Invitrogen PA5-22388) (Western blot only)

Species specific fluorescent secondary antibodies conjugated to AlexaFluor 488, 647 and 790 (all from ThermoFisher) were used at 1:500 for all experiments.

Validation

We have successfully used the following antibodies for immunostaining in previous manuscripts with a high level of success:
PMID 38419656: rat anti-SST, goat anti-PV, rabbit anti-nNos, rabbit anti-Olig2
PMID 19295152: rat anti-HA

Mouse anti-GFAP, from SigmaAldrich website: The antibody reacts specifically with GFAP in immunoblotting assays and labels astrocytes, Bergmann glia cells and chondrocytes of elastic cartilage in immunohistochemical staining. The antibody reacts with glial specific antigen in frozen or alcohol-fixed tissue sections.

Rabbit anti-NeuN, abcam website: Rabbit recombinant monoclonal antibody used for detecting NeuN, a neuronal marker. Validated for ICC/IF, western blotting, IHC and flow cytometry (Flow Cyt). Suitable for human, mouse, and rat samples. Cited in over 865

publications.

Rabbit anti-vimentin, Cell Signaling website: Vimentin (D21H3) XP® Rabbit mAb detects endogenous levels of total vimentin protein. Cited in over 3000 publications.

Mouse anti-MBP, ThermoFisher website: MA1-10837 detects Myelin Basic Protein from human, non-human primate, rabbit, sheep, goat, rat, and mouse samples. MA1-10837 has been successfully used in ELISA, Western blot, and immunohistochemistry applications. Images from rodent brain immunostaining on webpage.

Rabbit anti-H3K4me3, Cell Signaling website: Antibody detects endogenous levels of histone H3 when tri-methylated on Lys4. This antibody shows some cross-reactivity with histone H3 that is di-methylated on Lys4, but does not cross-react with non-methylated or mono-methylated histone H3 Lys4. In addition, the antibody does not cross-react with methylated histone H3 Lys9, Lys27, Lys36 or methylated histone H4 Lys20.
sdf

Rabbit anti-H3K4me1, abcam: Anti-Histone H3 antibody (Mono Methyl-K4) is a rabbit polyclonal antibody that is used to detect Histone H3 in western blotting, IHC, ChIP and ICC/IF. Suitable for human, mouse and rat samples and more. It's cited in over 1,100 publications.

Rabbit anti-H3K4me2, abcam: Rabbit Polyclonal H3 di methyl K4 antibody. Suitable for ICC/IF, ChIP, WB. It's cited in 320 publications.

Mouse anti-Ki67, BD Biosciences: Monoclonal antibody Ki-67, applications include flow cytometry & immunohistochemistry, cited in ~900 publications.

Rabbit anti-PH3, ThermoFisherPhospho-Histone H3 (Ser10) Antibody detects endogenous levels of histone H3 only when phosphorylated at Ser10. This antibody is not cross-reactive with other phosphorylated histones or with acetylated histones.

Rabbit anti-Cux1, Proteintech: Per website, antibody has been successfully used ifor immunostaining in dozens studies to label Cux1 in a variety of tissue.

HumanFAB rhodamine anti-Tubulin, BioRad: This antibody is specifically designed for use with the Chemidoc Imaging system for Western Blots.

Rabbit anti-H3.3, Invitrogen: Per website, this antibody has undergone advanced verification to confirm its efficacy and specificity for use as a pan-H3.3 antibody in numerous applications.

Animals and other research organisms

Policy information about studies involving animals; ARRIVE guidelines recommended for reporting animal research, and Sex and Gender in Research

Laboratory animals	The following mouse trains were used, all on a C57/B6 background: LSL-K4M (gift of Dr. Kai Ge, NIDDK); Nkx2.1-Cre (Jax# 008661); Lhx6-Cre (Jax# 026555), Ai9 (Jax# 007909), Sun1-sfGFP (Jax# 030952). Mice ages used for all experiments ranged from E13.5 through 12-months-old. Mice were housed under standard conditions (12h light and 12h dark). The morning on which the vaginal plug was observed was denoted E0.5.
Wild animals	N/A
Reporting on sex	All single cell Multiome analysis was performed separately on males & females for each age, tissue and genotype (See Supp Table 1). All behavior assays were performed on separate cohorts of males and females for all genotypes (see Data Sheet 2). We highlight differences between male and female mice regarding body weights, cell counts, gene expression analysis and behavior assays throughout the manuscript. We did not distinguish between males and females for many analyses of embryonic tissue (e.g., Figure 1), nor for some immunohistochemistry or in situ hybridization experiments as noted in the manuscript.
Field-collected samples	N/A
Ethics oversight	All experiments were approved by the NICHD Animal Care & Use Committee (ACUC) under protocol# 23-047.

Note that full information on the approval of the study protocol must also be provided in the manuscript.

Plants

Seed stocks	N/A
Novel plant genotypes	N/A
Authentication	N/A

Flow Cytometry

Plots

Confirm that:

- [x] The axis labels state the marker and fluorochrome used (e.g. CD4-FITC).
- [x] The axis scales are clearly visible. Include numbers along axes only for bottom left plot of group (a 'group' is an analysis of identical markers).
- [x] All plots are contour plots with outliers or pseudocolor plots.
- [x] A numerical value for number of cells or percentage (with statistics) is provided.

Methodology

Sample preparation	For all 10x Genomics Multiome reactions, Single nuclei dissociations from E13.5 MGE, E13.5 hypothalamus, P60 MGE-derived cortical interneurons and P60 hypothalamus cells from Nkx2.1-Cre;H3.3K4M;Sun1-eGFP mice were prepared as previously described in our STAR Protocols paper (PMID: 36520627), and described in detail in the Methods section of our manuscript. For each brain regions/age/sex, samples from multiple mice were combined to increase cell numbers. Draq-5 was added to single nuclei dissociations for positive selection of nuclei. We collected GFP+ nuclei from WT, Het and Hom mice, as these are Nkx2.1-lineage cells expressing the desired mutation.
Instrument	Sony SH800 cell sorter
Software	SH800 software
Cell population abundance	E13.5 MGE: ~80-90% of nuclei are GFP+. E13.5 Hypothalamus: ~40-50% of nuclei are GFP+. P60 MGE-derived cortical interneurons: ~5-9% of nuclei are GFP+. P60 Hypothalamus: ~25-30% of nuclei are GFP+.
Gating strategy	Initial gate was FSC-A vs SSC-A to enrich for nuclei over debris. These positive gated cells were then applied to a Draq5-A vs. FSC-A to select for Draq5+ nuclei. Singlets vs. doublets could be distinguished in this plot, so we selected specifically for the singlet Draq5+ nuclei population. These gated nuclei were then applied to an EGFP-A vs. FSC-A plot, where there was a clear shift (>1+ order of magnitude) of GFP+ nuclei along the EGFP-A axes, allowing us to collect GFP+/Draq5+ nuclei. Extended Data Figure 7 shows our gating strategy and examples of sorted cells.

- [x] Tick this box to confirm that a figure exemplifying the gating strategy is provided in the Supplementary Information.
